# Editorial: Coping With the Pediatric Coping Literature: Innovative Approaches to Move the Field Forward

**DOI:** 10.3389/fpsyg.2022.885679

**Published:** 2022-03-29

**Authors:** Line Caes, C. Meghan McMurtry, Christina L. Duncan

**Affiliations:** ^1^Division of Psychology, Faculty of Natural Sciences, University of Stirling, Scotland, United Kingdom; ^2^Department of Psychology, University of Guelph, Guelph, ON, Canada; ^3^McMaster Children's Hospital, Hamilton, ON, Canada; ^4^Department of Psychology, West Virginia University, Morgantown, WV, United States

**Keywords:** coping, pediatric, chronic conditions, chronic illness, childhood and adolescence

## Introduction to Coping

Chronic illnesses, injuries, and other health conditions (herein “conditions”) such as sickle cell disease, chronic pain, and burns are life-disrupting challenges for children and their families. Coping strategies can be defined as “cognitive and behavioral efforts to manage specific external or internal demands that are appraised as taxing or exceeding the resources of a person” (Lazarus, 1991, p. 112). In the context of chronic pediatric health concerns, children and their caregivers/parents must cope with the management of the condition itself, its indirect impact and associated treatment on their daily life (e.g., effect on school engagement), in addition to unrelated “everyday” stressors (e.g., parenting, peer conflict) (Turner-Cobb, 2013). Despite a substantive body of literature exploring coping strategies and quality of life in children living with a chronic condition, several theoretical and empirical gaps remain, including a large number and variable application of coping frameworks or models together with vague and inconsistent operationalization of coping strategies. For instance, Rudolph et al. (1995) proposed a conceptualization of coping that distinguishes between coping responses, goals, and outcomes. Coping responses are actions initiated in relation to a perceived stressor, while the goals are the reasons behind the engagement in a coping response, and the outcomes are the consequences of the coping response. Yet, these different components of coping have been used interchangeably in the context of pediatric chronic health conditions, with assessment or conceptualization of each aspect of coping varying substantially within and across health concerns. Consequently, the goal of this Research Topic “*Coping with the Pediatric Coping Literature: Innovative Approaches to Move the Field Forward”* was to bundle innovative and cutting-edge research that increases our understanding of coping strategies and their underlying mechanisms within pediatric chronic health conditions.

## Coping with the Pediatric Coping Literature

The Research Topic contains nine original articles illustrating the variety of innovative work being conducted around pediatric coping. This Research Topic of articles covers a wide range of populations [i.e., pediatric burn survivors (Snider et al.), chronic pain (Balter et al.; Caes et al.; Nabbijohn et al.; Wakefield et al.), spina bifida (Ohanian et al.), sickle cell disease (Hood et al.), inflammatory bowel disease (Reed et al.), and Median Arcuate Ligament Syndrome (Stiles-Shields et al.)], across various methodologies [i.e., cross-sectional questionnaire studies (Snider et al.; Hood et al.; Reed et al.), longitudinal evaluations (Ohanian et al.), diary assessments (Caes et al.), scoping reviews (Nabbijohn et al.), and qualitative explorations (Wakefield et al.)]. Including a focus on children and their parents, these articles highlight the important role many coping responses [e.g., concealment (Wakefield et al.), primary control coping (Ohanian et al.), distraction (Snider et al.), catastrophic thinking (Caes et al.; Reed et al.)] play in understanding how a pediatric chronic condition can impact a child's functioning and wellbeing, as well as the need to adopt an integrative biopsychosocial approach to the assessment, formulation, and management of pain coping that goes beyond focusing on pain intensity (Hood et al.). Beyond expanding the theoretical understanding of pediatric coping mechanisms, two articles within the Research Topic also explored interventions to support the uptake of adaptive coping skills (Balter et al.; Ohanian et al.). In particular, these studies highlight how interventions aimed at coping responses are feasible, even in a population with high prevalence of comorbid psychiatric disorders (Stiles-Shields et al.), and can lead to improvements in pain experience, socioemotional functioning, and psychosocial inflexibility (Balter et al.). Nabbijohn et al. present a comprehensive scoping review that gives an overview of the state of the art of the coping literature in the context of pediatric chronic pain. This review highlights four current challenges the pediatric chronic pain coping literature faces, and provides clear suggestions on how to overcome current gaps in the field: (a) lack of theory, conceptual clarity, and conceptual consistency across research; (b) need for diverse methodologies, along with better consistency or standardization in measurement across research; (c) inclusion of diverse populations to understand variations in coping approaches; and (d) improved conceptualization and measurement of proactive coping through theory testing/validation and revision. Arguably, these gaps are reflected in the pediatric health coping literature more broadly.

The Research Topic highlights key questions that remain for future research, aligning with trends seen across the broader pediatric coping literature. Firstly, the research focuses on coping *responses* and their *outcomes*, with little known about coping *goals* or the reasons why children and/or parents engage in these coping responses. A better understanding of the reasons or motivations to engage in a particular coping response is crucial for a comprehensive understanding of the benefits of a coping response and how these benefits could be situationally specific (Carver and Scheier, 2001). Secondly, due to the predominant focus on coping by the child, there is a clear need for family-based assessment of coping processes accounting for the dynamic and interrelated coping of family members. Thirdly, all articles focus on coping with condition-related issues and their indirect impact on daily functioning, but none examine coping with everyday stressors unrelated to the health issue. As human beings with multiple roles, responsibilities, and facets to our identity, we need to adopt a correspondingly integrative view of coping. Lastly, most articles explore potentially harmful coping responses (e.g., concealment, catastrophic thinking). This over-focus on risk factors parallels most research also targeting specific conditions, such as chronic pain. The combined examination of risk and resilience factors is an important avenue for future research, and various models, such as the Resilience-Risk Model for Pediatric Chronic Pain (Cousins et al., 2015), have been put forward to stimulate such research and inform treatment approaches.

## Author Contributions

LC drafted the first version of the editorial, with CMM and CLD providing feedback and additions on structure and content. All authors approved the final version.

## Conflict of Interest

The authors declare that the research was conducted in the absence of any commercial or financial relationships that could be construed as a potential conflict of interest.

## Publisher's Note

All claims expressed in this article are solely those of the authors and do not necessarily represent those of their affiliated organizations, or those of the publisher, the editors and the reviewers. Any product that may be evaluated in this article, or claim that may be made by its manufacturer, is not guaranteed or endorsed by the publisher.
